# Barriers to formal health care seeking during pregnancy, childbirth and postnatal period: a qualitative study in Siaya County in rural Kenya

**DOI:** 10.1186/s12884-019-2485-2

**Published:** 2019-09-18

**Authors:** Caroline A. Ochieng, Aloyce S. Odhiambo

**Affiliations:** 1 Stockholm Environment Institute, Linnegatan 87D, Box 24218, 10451 Stockholm, Sweden; 2 Ryan Institute, National University of Ireland Galway, University Road, Galway, H91 REW4 Ireland; 3 Safe Water and AIDS Project (SWAP), P.O.Box 3323, Kisumu, 40100 Kenya

**Keywords:** Antenatal care, Facility delivery, Home birth, Postnatal care, Qualitative study, Maternal mortality, Kenya

## Abstract

**Background:**

There is broad agreement that antenatal care (ANC) interventions, skilled attendance at birth and management of complications arising after delivery are key strategies that can tackle the high burden of maternal mortality in sub-Saharan Africa. In Kenya, utilisation rate of these services has remained low despite a government policy on free maternal care. The present study sought to understand what factors are leading to the low healthcare seeking during pregnancy, child birth and postnatal period in Siaya County in Kenya.

**Methods:**

Six Focus Group Discussions were conducted with 50 women attending ANC in 6 public primary healthcare facilities. Participants were drawn from a sample of 200 women who were eligible participants in a Conditional Cash Transfer project aimed at increasing utilization of healthcare services during pregnancy and postnatal period. Interviews were conducted at the health facilities, recorded, transcribed and analysed using thematic analysis.

**Results:**

Multiple factors beyond the commonly reported distance to health facility and lack of transportation and finances explained the low utilization of services. Emergent themes included a lack of understanding of the role of ANC beyond the treatment of regular ailments. Women with no complicated pregnancies therefore missed or went in late for the visits. A missed health visit contributed to future missed visits, not just for ANC but also for facility delivery and postnatal care. The underlying cause of this relationship was a fear of reprimand from the health staff and denial of care. The negative attitude of the health workers explained the pervasive fear expressed by the participants, as well as being on its own a reason for not making the visits. The effect was not just on the woman with the negative experience, but spiraled and affected the decision of other women and their social networks.

**Conclusions:**

The complexity of the barriers to healthcare visits implies that narrow focused solutions are unlikely to succeed. Instead, there should broad-based solutions that focus on the entire continuum of maternal care with a special focus on ANC. There is an urgent need to shift the negative attitude of healthcare workers towards their clients.

**Electronic supplementary material:**

The online version of this article (10.1186/s12884-019-2485-2) contains supplementary material, which is available to authorized users.

## Introduction

Maternal mortality—the death of women during pregnancy, childbirth, or within 42 days after delivery—remains a major public health challenge in low resource settings. The Millennium Development Goals (MDGs) called for 75% reduction in the maternal mortality ratio (MMR) by 2015 from 1990 levels [1]. Although considerable progress was made in reducing MMR under the MDGs, from 385 to 216 deaths per 100 000 livebirths which correspond to a relative decline of 43.9% [2], the progress differed greatly between regions. The MMR is still alarmingly high in sub-Saharan Africa, where many countries still have more than 500 maternal deaths per 100,000 livebirths [3]. Even within countries, there are significant regional differences. While MMR rates in Kenya stood at 360 maternal deaths per 100,000 livebirths in 2010, in some regions in the country the rates were 695 maternal deaths per 100,000 livebirths [4].

The Sustainable Development Goal (SDG) aims at less than 70 maternal deaths per 100,000 livebirths globally by 2030 [5]. According to Alkema et al. (2015) [2], understanding the drivers of progress in reducing maternal mortality—as well as the factors impeding progress—is key to making informed decisions for reducing the MMR in the post-MDG era. Evidence-based clinical and preventative interventions aimed at reducing maternal and neonatal morbidity and mortality are well documented. There is a general agreement that antenatal care (ANC) interventions, skilled attendance at birth and management of complications arising after delivery are key strategies that can tackle this burden [6–11].

Despite this consensus, utilisation of these health services has remained low in most of the affected regions. According to UNICEF’s 2014 estimates, only 48% of women in sub-Saharan Africa had their deliveries in health facilities. While this might appear to be in contrast to ANC where 71% of pregnant women are reported to attend formal ANC at least once, only 44% of the women come for the WHO recommended 4 visits [12–15]. Furthermore, most women who go for ANC are likely to wait until the second trimester for their first visit, while a substantial proportion present only in the third trimester [16, 22]. At this stage it is too late to identify potential complicating conditions and to prevent and/or manage them in time.

Numerous barriers have been documented that prevent women in low income countries from making the recommended health visits. Utilisation of health services have been linked to maternal and spouse’s education, marital status, income, media exposure and history of obstetric complications in systematic reviews [17, 18]. Most of the studies identified in the reviews have been quantitative studies that correlate healthcare seeking with selected variables and use these to draw statistical associations on the key determinants. These type of studies have been criticised for offering very little insight into how and why these factors influence the visits [13, 19, 22]. In turn they can lead to narrow framed policies such as provision of transport for women to make health visits or removal of service fees, while neglecting the underlying cultural and behavioural factors that underpin the apparent reticence towards healthcare seeking.

In Kenya, government intervention on maternal health has focused on user fees, which were abolished in 2013. Yet in a Kenya National survey [34], the largest share of respondents (42%) reported that delivering outside a health facility was because of the distance to the facility and lack of transport. Only 17% cited the fees levied at the facilities as the key barrier. With the exception of Nairobi where the ranking of user fee as a barrier to facility attendance was above 30%, the effect was negligible in other regions. Although the policy measure has been commended [35], others have argued that Kenya’s free maternity program is likely to have the biggest effect in Nairobi, a region which already has the highest rate of births delivered under medical professionals at 89%, compared to only 26% in the rural regions [36].

The present study sought to understand what factors contribute to the low healthcare seeking behaviour during pregnancy, child birth and postnatal period. The study sought to understand the challenges with health seeking in the entire maternal care continuum, so that future programs can be better informed on what factors to pay particular attention to when designing interventions. To be able to get an in-depth understanding into these factors, which can be quite complex given the cultural complexity that surrounds pregnancy and childbirth, we utilized a qualitative approach to data collection and analysis. The reporting of the study methods and results follows the consolidated criteria for reporting qualitative studies (COREQ).

## Study setting

The study was carried in Siaya Country, which is located in the shores of Lake Victoria in Western Kenya. The County is mostly rural, with a population of 8.3 million people which was projected to grow to 9.6 million by 2017 [20]. This is due to the high fertility rate of 5.5 children per woman compared to a national average of 4.6 children per woman. Nearly three quarters of the population is under 30 years old, and 45% is under 15 years.

Siaya County performs poorly and worse than national average for several development and health indicators. It’s Human Development Index (HDI) score is 0.46 against a national average of 0.56 [21]. It has the highest HIV (20% vs. 6% national rate), tuberculosis and malaria rates in Kenya; as well as the worst indicators of child and overall health status [20]. Infant mortality rate is 111 per 1000 live births and maternal mortality rates are 695 per 100,000 live births; against Kenyan national average of 49/1000 and 488/100,000 respectively [20, 37]. Thirty eight percent of the population live below the poverty line, which is slightly lower than the national average of 45% [21].

Although there is a high rate of ANC attendance in the County with 71% of the women making at least one ANC visit, the visits are often made very late in pregnancy, often in the third trimester [21]. Most women therefore miss the 4 recommended visits. Nearly half of all births in the County take place without skilled attendance and barely half the women come for postnatal visits [20]. ANC, delivery under skilled attendance and postnatal care within 42 days after birth are proven strategies for tackling the pregnancy related risks. Therefore, unearthing the barriers to care seeking along this continuum can lead to design of solutions that can alter the negative maternal and child health trends witnessed in Siaya County and other similar settings.

## Methods

### Participant recruitment

This study was part of a broader pilot study investigating the role of conditional cash transfers (CCTs) in motivating health facility visits for ANC, delivery and postnatal checks in Siaya County. The results of this qualitative phase was meant to inform the design and delivery of an intervention to tackle the identified barriers to health visits in the County, which is currently underway [39].

Participants were drawn from pregnant women attending ANC at public primary health care facilities in Boro Division in Siaya County. Boro is one of the four Divisions in Siaya, and was purposively selected for the study by recommendation of the County Health Committee because it exhibited the lowest service utilisation rates by pregnant women in Siaya County. Recruitment was done in six healthcare facilities, from where we drew a random sample of 10 women per facility, giving a total sample size of 60 women. Enrolment in the facilities was carried out by incentivized health staff with a research staff supervising the process. The enrolment was continuous and ceased when the target sample size for each facility had been accomplished.

Criteria for enrolment were that the woman had a pregnancy of 6 months or less, was a long-term resident of Boro Division (6 months and above) and had no plans to relocate in the next 12 months. Due to the nature of the intervention study whereby cash was being transferred to participants through mobile phone wallets (*M-pesa*), an additional enrolment criterion was for the women to have access to a mobile phone. The phone could belong to them or to a member of their household or anyone else they trust. There is very high mobile phone penetration in Kenya, and this criterion was easily met by all the women who were approached as potential participants. There were no refusals to participate in the study.

### Data collection

The qualitative data was collected using focus group discussions (FGDs) that took place in August 2015. Out of the 60 recruited women, 50 participated in the FGDs. In total, there were six FGDs, each one comprising of between 7 and 10 participants. Those who did not participate had either moved from the study location, miscarried or were untraceable. The FGDs were administered in the local language (Luo) by two local researchers who are familiar with the setting and moderated by the Field Coordinator for the study (ASO). The design of the data collection tools (FGD guides) was coordinated by the Principal Investigator (CAO) who is also conversant with the local sociocultural context and language; with further input from the research advisory team. CAO trained the local researchers on how to use the FGD guides and led the piloting of the tools.

All the FGDs took place at the health facilities where the women were enrolled, which tended to be their nearest health facility and the one they would seek most of their care from. The six health facilities were Segere, Kadenge, Kaluo, Karuoth, Boro and Nyathi. The health facilities were the only neutral and confidential venues available in the village where the women felt comfortable gathering in. The healthcare staff facilitated venue arrangements for the discussions but were not present during the interviews. Refreshments were provided to participants before commencement of the discussions which lasted for about 1 h. Participants also received a transport reimbursement of Ksh. 300 (USD 3).

Prior to the FGDs, the moderators reiterated the details of the study and the aim of the FGDs as well as what taking part in the FGDs meant for the women individually and as a community. This was done to ensure that the women fully understood what they consented to. They were then asked to sign consent forms if they were happy to proceed with the FGDs. They all opted for oral consent. The moderators therefore read out the contents of the consent form and the participants verbally agreed to the contents and to take part in the study.

The FGDs were conducted using a loosely structured question guide (Additional file 1) that had been piloted with 5 pregnant women in Sigomre health centre, a health facility in Siaya county but outside Boro division where the study was being conducted. The piloting was done to ensure that the final FGD guides had clarity and sensitivity to cultural nuances about health and personal life issues. All the FDGs were audio-recorded with the respondents’ consent. These were complemented with notes taking on key issues that emerged during the discussions. At the end of each FGD, one of the moderators summed up the key issues discussed in the sessions as part of data validation and verified with the respondents their take on the proceedings as part of data quality assurance. The moderators also sought participants’ permission to contact them at a later date in case there was need for clarification of some issues they raised or additional information during data analyses. All the participants consented to follow up contact and also to be involved in verification of the findings. After each FGD, the moderators held debriefing sessions to reflect on the process, share their observations and identify ways of improving the process in the subsequent FGDs.

### Data analysis

Data coding and analysis was carried out by the CAO, under the guidance of a research advisor (JAO). The analytical approach used for this paper was largely transcript and notes based thematic analysis [23, 24]. Data analysis started with familiarization through listening to the audio tapes several times followed by verbatim transcription of each FGD audio tape. As Krueger and Casey [23] have noted, it is important to listen to someone’s story and understand it well before telling it, because that is what analyses of focus group data entails. The transcripts were then re-read repeatedly, noting down any points that stood out from the initial readings.

The transcripts were then subjected to coding, initially assigning descriptor statements to text that presented particular meanings. This was done for each of the FGD transcripts and then compared across the transcripts. The process entailed breaking down the data and reassembling it together through constant comparison [24] of transcripts and notes to tease out underlying meanings. Emerging themes were identified, checked to see if they corresponded with the extracted data linked to the codes attached to them, and if they fitted the entire data from the transcripts and the notes. After retrieving all the emerging themes, they were then redefined and renamed to develop clear story lines from the data. These themes and related issues were also compared with data from literature to check for similarities and differences. For example, barriers to ANC attendance reported by the present participants such as distance to facilities and financial constraints were checked against reports from related research from other low resource settings.

## Results

The characteristics of interviewed respondents are summarised in Table 1. The average age was 23 years. Participants had 3 children on average, and initiated ANC at 24 weeks as per the health records. The ANC records are usually based on the women’s own self-reporting of their last monthly period (LMP). This measure is however inaccurate, with many deliveries reported to have occurred before the expected delivery dates. Education levels were low (less than 20% with secondary level education), so was socio-economic status as measured by the type of floor of the house. Most of the women reported being married.

**Table 1:** Characteristics of study respondents

Health Facility of interview	Age	Pregnancy stage at 1st ANC visit (weeks)	Gravidae	Marital Status		Primary means of travel to facility		Educational attainment		House floor type	
				Married	Single	Foot	Car or motorbike	Primary	Above primary	Mud	Cement
Boro	21.4	28	1.6	5	2	0	7	5	2	6	1
Kadenge	24.3	22.8	3.2	7	1	1	7	5	3	5	3
Kaluo	24.4	24.2	3.1	7	1	2	6	5	3	6	2
Karuoth	21.5	20.5	2.4	10	0	3	7	8	2	7	3
Nyadhi	20.5	23	1.8	6	3	1	8	9	0	8	1
Segere	28.1	24.5	4.1	8	0	2	6	6	2	8	0
Total	23.4	23.8	2.7	43	7	9	41	38	12	40	10

Nine main themes were identified which addressed the central research question on the barriers to healthcare seeking. While some themes cut across all the domains of care (ANC, facility delivery and postnatal visit), some were specific to a particular stage of the health care continuum. The following major themes were evident: 1) *benefits of making the visit* for own health and that of the un-born baby; 2) *means of transportation*, including availability during emergency (at onset of labour), affordability and suitability (for postnatal visit); 3) *embarrassment* of how one is perceived by peers and staff; 4) *medical interventions* and perception of risk as per prior individual experience or of others; *5) financial cost of visits* and 6) *autonomy in decision making*. For facility delivery, the following additional themes were identified: 7) *attitude of health workers* from personal or narrated experiences and *8) unpredictability of labour.* The *health status after delivery* (9) was a recurrent theme in post-natal visits. The themes themselves were interlinked in multiple ways, with others very distinct (7, 8, 9) and others having very strong overlaps. Some themes also gave rise to important sub-themes such as procrastination (from theme 1), financial empowerment (5 and 6), denial of care and punishment for late initiation of visits (7), and role of Traditional Birth Attendants (7).

### Perception on health visits for ANC, delivery and postnatal care

The importance of the health visits was clearly acknowledged by participants. Treatment of general illnesses in the mother was viewed as an important role of ANC, and prevention of transfer of some of these ailments to the baby was also emphasised. HIV detection and treatment was mentioned, but only through prompting. Otherwise specific ailments cited were malaria, body aches and breathlessness.

The following statements exemplify the importance of ANC visits for treatment.

*Going to clinic is good because at times you have ailments, for example I usually get very bad stomach pains (when pregnant) that even walking is something I have to strain myself to do ..when I go to clinic they find how to help me early on – P7F1*

*When pregnant am always sick, for 3 months am sick. I therefore must always go to clinic so they can find out what is ailing me – P8F1*

*The reason people go to clinic is to receive help with illnesses that people get. Once you have been tested you can get help and treatment so that those illnesses do not also get transferred to the baby - P4 F5.*

*It is good (going to clinic) because of the injection you receive; the injection works in your body and helps you. When you deliver, it finds when the injection had worked in your body – P7F4*

The results were however mixed on the importance of test results. While others cited HIV test amongst the important health interventions that made them go for ANC, others observed that getting to learn about their HIV status would be a reason for not making ANC visits.

*There is embarrassment that your blood will be tested, and they will find something in your blood, therefore you feel embarrassed that you are still young and you will be told something (positive HIV test results after prompting) that will shock you – P7F2.*

*…..on the first visit they must test for your status (HIV after prompt), and this brings fear a lot. After this first visit it is very easy to make the next visits – P2F3.*

Participants also attached importance to checking of the baby’s lying position and early detection of complications in the baby.

Unlike ANC visits, only a few observations were made with regard to the importance of facility delivery and postnatal care. Importance was attached to facility delivery for emergency care if delivery turned out to be complicated, which they observed could not be predicted beforehand.

*It is good to deliver at a hospital, delivery can go badly, and in hospital you can be helped* - *P2F2*

The perceived importance of emergency obstetric care was manifested in ANC visits that some women only attended in order to ensure they can have facility admission for delivery should complications arise at delivery.

Although postnatal visit was acknowledged as important, not much was articulated in terms of its importance.

### Challenges of making health visits

The participants described a number of factors that deter making the initial health visit and honouring subsequent appointments as discussed below.

#### ANC

Participants reported multiple challenges for ANC visits of which distance to health facilities and lack of transport were the most recurrent. The pregnancy state of the woman made it challenging to walk the long distance to the health facility, and to utilise the common mode of transport which is motorbike taxis. This challenge was augmented by lack of finances to pay for the transport.

*You know that in this area the dispensaries are not very close.... To go to the nearest dispensary you must use fare. You are tired. So if you lack that fare, you decide that you will start going to the clinic later ..it makes you lazy - P1F2*

*There is no reason to start (going to clinic) early. You start in ‘late hours’. If you start early you will walk ‘for a long time’. It is better starting with 8 months or 7 months (pregnancy stage) – P3F3.*

Fear of embarrassment was another commonly cited reason for not making the health visits, with different underlying factors. Young people who had their first pregnancy would be embarrassed to go to the clinic and be seen. There was also embarrassment from having pregnancies closely following each other. The fear was from peers and from health staff.

*You might give birth, and then by bad luck you become pregnant again in a very short time, a pregnancy on top of another …you will fear going to the clinic because someone will ask “so and so who we were with in clinic the other time is back again?” This can cause stress and embarrassment and make you not go to the clinic – P9F1*

*You may have given birth to the first child. And then you get the second pregnancy when (pause), as in, you have not planned well. When you come to clinic the Sisters (health staff) will talk to you badly. “What sort of rush is this?” - P3F2*

Another commonly cited reason for not making the visits was *Samuoyo*, which is a local terminology with no specific English translation, although it relates to procrastination and laziness.

*Some people have ‘Samuyo’ which prevents them from making the visits. When it is time for their clinic visits they miss going - P4F1*

*Please elaborate on what you mean by Samuoyo. What exactly is preventing them from making the visits? ……..Interviewer*

*… (Silence).. – P4F1*

This reason was however almost always coupled with long distance to health facility, lack of transport, money and other more specific factors that hindered the clinic visits as exemplified in the statement below.

*…you come from far (distance from clinic), and you have a back ache ailment, the day you need to go to clinic you are feeling back pain, and you get Samuoyo and plan to go tomorrow, and tomorrow comes and you don’t go - P9F4*

Fear of learning one’s HIV status was also stated as a deterrent, although as stated earlier, this contrasted with other views that welcomed the tests and in fact cited it as a motivation for attending ANC. Similarly, there were contrasting views with on other ANC interventions such as feeling of the baby by the health staff, number of tests and probing questions from health workers.

Other reasons mentioned as deterrents but less recurrent were busy household schedules, lack of appropriate clothing to wear and local church doctrines that forbid ANC.

Often, the challenges were reported as making the women postpone making the visit, rather than making a choice of never making the visit. In fact, the participants never really decided against going to ANC for lack of transport and other financial related factors. Rather, they procrastinated (experienced *Samuoyo*) until it was too late for a group of them, who would have had their deliveries. For others, they would procrastinate until there was a stronger motivation for making the visit beyond the routine treatment of ailments. The motivation was often the insurance for facility delivery, which required one to have had previous contact with the health facilities through ANC.

The observation below is an illustration of this, very similar to the observation made by *P9F4* and others:

*Clinic is far, and you are sick, sick all the time…you are also coming from far, so you just keep telling yourself you will go tomorrow and you miss, and the days keep going, until it is too late, the baby has arrived! – P4F2.*

#### Facility delivery

Participants constantly cited the unpredictability of labour commencement and its inconvenient timing as a reason for home delivery.

Participants reported that labour tends to begin at night when there is no means of transport available to the nearest health facility. This challenge was coupled by the long distance to the health facilities that operate at night and weekends, lack of funds and inaccessibility of the roads at night when it rained. They would thus have no option but to deliver at home. Others observed that at times they would try to “hold” until morning but would then end up with a home delivery. The decision to deliver at home was not often the preferred choice for these participants, and there was a constant narrative of “being forced to deliver at home”.

*Sometimes labour has started while you are at home, and the hospital here (F5) does not operate at night, so you have to go to Siaya (the District hospital) but then you have no means of going to Siaya, so you are forced to deliver at home - P6F5*

*Delivery is not something predictable. At times you have been told at the clinic that it will occur next month or end of next month, and that is what you have known all along. Then it (labour) comes at a time you never expected! It has rained, the road is bad, and even if you have money you will not find anyone willing to take you to the hospital – P1F2*

*….it has started, you indeed feel it, the pain!, but you have no money in the pocket, and whoever you are going to ask money from cannot give it to you on the spot, and there is actually no one you are going to wake up and ask money from or ask to transport you to hospital – P9F1*

*…labour has started and it is a Weekend. F1 is close and you can go there immediately but it is closed on weekends, so you now have to figure out how to go to Siaya, you risk delivering on the way to Siaya, it is therefore better if you deliver at home – P7F1*

Lack of realisation that one was in labour was also cited as a reason for home delivery by some participants. They reported difficulty in distinguishing between false and real labour, or if they were having labour at all. This led to several outcomes all of which eventually contributed to a home delivery. The women either waited too long before going to a health facility and therefore delivering at home. Others went in too early and were turned away, therefore chose to deliver at home because they could not afford the cost of making another journey to the facility. For other women who went in early, the experience they had and services they received were not desirable. The undesirable experience was narrated to other women, who would in turn not want to make the journey early.

*… Sometimes you have gone in early and your delivery time has not arrived. Before you realise what is happening the Sister comes in with scissors, they want to cut you (Caesarian section) – P11F4*

*You can go in for delivery, at times your time for delivery has not reached. You are going to suffer! The doctor is slapping your thighs…. It completely turns you off going to deliver in a hospital - P1F5*

*You are aware that there are some babies that do not cause any pain; … after having no pains you suddenly have it and think the labour is just beginning, but then baby has already come out! So there is no way you could have gone to the hospital – P1F6*

The attitude of health worker was a prominent determinant of home delivery. There was fear of mistreatment by health workers that included being verbally abused, beaten, and being left unattended. The health worker attitude not only influenced the decision of women who had lived through the experience, but also those who had heard stories of “what happens in those places” *(P6F1, P4F1, P5F4).* The negative attitude of health workers was in direct competition with the sympathetic, supportive and caring attitude of traditional birth attendants (TBAs).

*There are some health workers within those hospitals who have very bad character. True!, there are some really bad people there, they make mothers fear going to those places – P4F1*

*People tell you what Sisters do to people there, how people are beaten there, so you get scared and say to yourself “am not going to that place where am going to be beaten. I would rather just give birth at home” – P5F4*

*Some sisters are very harsh, when you are in labour instead of soothing you, they just become harsh towards you, instead of being patient with you she is harsh and quarrelling you….. (emotional); personally when I went to deliver in a certain facility in ……(name of a facility), I saw when the sister suddenly became very harsh, then I started getting fearful; so later when you think of that fear you experienced you say aah, let me just give birth at home – P2F4*

*The TBA treats you well, talks to you well, at times you have already given birth to two or three children. While if you go to Siaya (hospital) you get trainees or a doctor who shouts “Spread your legs wide! I did not send you (to get pregnant)!”. So when you imagine that mistreatment and those words, you decide its better you look for that TBA who treats you well and talks to you well – P8F6*

Previous ANC visits was also a determinant of home delivery. Participants reported that they feared they would not be admitted for delivery if they had no ANC book, or if they had missed many ANC visits. There was fear of both denial of services but also reprimand from health staff for having skipped ANC visits.

*You did not go to clinic (ANC) and when you arrive (for delivery) the first thing you are asked for is the Card; it forces you to deliver at home – P8F6*

The type of health services provided also deterred facility delivery. Participants reported underlying perception that some of these medical procedures were unnecessary, and at times even punitive, and these would make women choose not to deliver at health facilities. The medical procedures that created fear included C-section, stitching and the medical instruments used in those procedures.

*When one goes to hospital for delivery, and at times one is scared, and she starts crying there, within that minute that she is crying they will take her to the knife (C-section), that is why they find it difficult to go (to health facility for delivery) – P4F4*

*In a hospital like Siaya, there is usually a list that you are told to fill in immediately you arrive, a list for operation (Surgery), it is filled in very very quickly, you get in and the first thing you are asked to do is to fill it in – P9F1*

*The instruments they use gives you fear. You arrive (at facility) and you see the instruments, and imagine that those instruments are going to be used on me! You decide that it is better to go and give birth at home – P6F6*

The medical importance of C-section was not perceived at all by some women as illustrated in the participant’s quotes below on reasons for choosing to have a home delivery.

*There is fear that if the baby is big you will be sent in for surgery - P4F3*

*At times one wants to deliver at the facility but fears having surgery. Sometimes you have been prescribed for surgery and you do not want to go for that surgery – P9F4*

*For example you had a sweet mouth while you were pregnant, you ate a lot of fruits, and most people have told you to stop eating fruits such as avocado because the baby will become big in your stomach and you will be sent in for operation, that can also make you fear going – P2F6*

Similar to ANC, fear of embarrassment made some women to opt for a home delivery. Embarrassment of being attended to and seen naked by a male staff was commonly cited. Other sources of embarrassment were lack of clothing for the baby to wear while going home from the hospital; the main concern being how the health staff and other people would react to this.

*You can also be embarrassed because you have not yet bought clothes for the new born, and that when you go to the hospital the doctors will talk to you badly, they will say “what were you doing all these months that you have not yet bought clothes for the baby?” P6F2*

Financial costs was yet another deterrent to facility delivery, but also underlie several other reasons stated as barriers to facility delivery such as lack of transportation and clothing for the baby. Additional financial barriers were lack of funds to cover for the cost of facility delivery. Despite the removal of user fees, the perception of being detained in hospital for lack of payment of user fees as used to occur in the past still prevailed. There were also concerns associated with other hidden costs of facility delivery.

*Before you go to a facility you assess how you live and it is not good; your husband does not have a good income; in the facility they will require a lot of money – P4F1*

*To emphasise what my sister (P4F1) has said, true, you really don’t have money; if it (delivery) finds you at home you can pick a ‘leso’ (linen wrapper) like this one (points) am wearing and you use it to very quickly cover the baby. But if it comes here (at the health facility) and you don’t even have anything for him to wear; will I leave with an unclothed baby? Won’t people laugh at me? So yes, less income is a problem – P9F1*

*The challenge of going to deliver at a health facility has to do with money. Even though you don’t pay for the delivery they require you to buy things for wrapping the baby – P1F3*

The challenges highlighted above were not only incorporated by the women in their decision making, but also by the people close to them. Participants reported that spouses and close families would also influence their choice to deliver at home.

*At times your husband says that just give birth at home because in the past women gave birth at home, they never went to give birth in hospital, and at that time you are already in labour, so you will be forced to deliver at home – P1F6*

From the discussions, it clearly emerged that financial decisions related to facility delivery were generally the men’s domain. From the spouses and family perspective (often mother in law), facility delivery was an unnecessary cost that in addition brought about substantial risks. The men were said to be particularly opposed to the high cost of discharging women from hospital after delivery and expressed the fear of their spouses getting detained in hospital for lack of user fees. The perceived costs and risks were often tied to and substantiated with very low or cost free alternative of a home delivery under the support of trusted and caring traditional birth attendants. Previous experience with a successful home delivery would determine a future home delivery.

*You may have given birth to the first child, the second and even the third child at home, therefore one feels that even the one they are carrying can also just be delivered at home, so they just stay home and plan to deliver at home like they had done with the previous ones – P2F5*

#### Postnatal visit

Some challenges for postnatal visit were similar to ANC and facility delivery challenges. Procrastinating was given as a reason for delaying the visits until the period had fully elapsed. Distance to health facility and transport challenges were also consistent with challenges experienced during ANC visits and facility delivery.

Some challenges were however unique to this stage of the maternal continuum of care. The commonly stated reason for not attending postnatal visits was the state of health of the mother after delivery that could not allow her to travel for a health visit. Many women reported they would be suffering from stomach cramps, bleeding, back ache, painful stitches and a variety of other ailments that made it impossible to walk to the health facility. For some women this pain would go on for months and they had to wait until they had recovered from the ailments to be able to make the visits. The available mode of transportation (motorbike taxi) was also stated as a problem for some women. There was fear of the baby falling off when utilising the motorbike taxi.

*After a home delivery, you are in such a mess! You don’t even have the foot to make any small step with, let alone walk to hospital; there is also pain that people get when they deliver at home – P9F1*

*Sometimes you have delivered, and the baby was so big he has torn you up. It is impossible to sit on a motorbike in that state and with an aching body. You must wait until you have healed – P3F2*

Fear of embarrassment was also reported, contributed to by several factors as exemplified in the statements below. As with ANC, the source of embarrassment is not just other people but also the healthcare provider; in terms of what they would think or say about the women.

*You may have given birth to a child who has no good health, therefore you feel embarrassed that this baby is still so small and with poor health, she will not produce any weight (on the weighing scale at the facility), so you decide to keep breastfeeding her for a while so she can gain some weight before you can take her to clinic – P2F2*

*You may not have found yet a good attire for the baby to wear, so you feel that if you go to the hospital the doctor will see the child ‘kofuchore’ (Unkempt) and the doctor may talk to you badly about it. Therefore, you take time until you can find proper clothing for the baby before making the visit – P1F2*

*At times you have given birth at home, the baby has a defect on the body and you do not want people to see this, so you want to keep the baby hidden in the house here and not take him/her out - P4F1*

*When I have given birth I normally bleed a lot, am therefore embarrassed leaving the home, for fear that the blood may overflow - P4F2*

From the narratives, missed ANC visits and home delivery was yet another deterrent for postnatal visits. Participants reported fear of reprimand by the healthcare workers for not having made ANC visits, and for delivering at home if they showed up at postnatal stage with the baby. There was also fear of being denied care.

*Here at F1, if you give birth at home they never allow your baby in for clinic (postnatal visits), you can be sent away with the baby, and because you know you are going to be sent away you do not bother to go – P6F1*

*You may have given birth at home, and you never used to go to clinic, you have no clinic card, so how will you even begin taking the child to the hospital and you never took the Card (ANC card)? So you are forced to just stay home - P2F6*

*The way I never used to go to clinic, and now I have delivered at home, and I want to take my baby for postnatal visit. The Sister will definitely ask why I never used to go to clinic. That gives me fear whenever I think of going back and that makes me not to go – P2F4*

The fear of reprimand from health workers cut across many of the other deterrents mentioned earlier such as lack of proper clothing for the baby to wear, failing to honour recommended visits or to follow the health provider’s recommendations from past visits, and for having a baby who has a low weight.

Like the ANC and delivery visit, the medical interventions were also a deterrent to postnatal visits. The fear of vaccination was constantly stated. The vaccine hesitancy was not only a concern coming from the women. In many of the narratives it was reported as an objection coming from family members: the spouses and the mothers in law. Unlike with the two previous stages, the family influence on the visits was particularly strong at the postnatal stage.

*The number of injections has increased these days, sometimes you come back home and the baby cries from all the injections, your husband then says “if I find you have taken the baby for those injections again you will see!” (Face consequences) – P3F3*

*It depends on how you live in the home with your mother in law. You may have taken the first child to the clinic who received a vaccination and then cried a lot afterwards. When you want to go back with the same or another child she tells you that “I never took the father of this baby for vaccination and he is alive to date and has sired this child …so why are you bothering us with this vaccination issue?” - P2F3*

*Some husbands refuse that children should not receive injections, that injections cause pain, he says that his mother grew up without being taken to hospital for injections and she is still alive to date, so after he tells you that you also decide not take the child to the clinic – P4F4*

*After you have taken the baby to the clinic and she has been given an injection, it gets a bad swelling, and the baby feels pain ….whether I take the child to the clinic or not I have already given birth – P4F6*

Other deterrents mentioned were long waiting times queues at the facility, fear of reprimand for failure to follow the instructions of health worker from previous visits, going in and finding the services are not available and to have to make the journey again, and lack of the spouses’ support in paying for transport.

## Discussion

This study sought to understand the barriers to healthcare seeking during pregnancy, birth and postnatal period among pregnant women in rural Western Kenya. The study was conducted in a setting that has very high mortality rate of over 600 deaths per 100,000 live births, and low utilisation of available health services for maternal care. A qualitative approach was employed to unearth insights on factors underlying the low service utilisation.

The study generated key insights on the underlying determinants of ANC visits, facility delivery and postnatal visit. Previous studies have mainly reported economic reasons such as transport, education and employment status of the woman as key barriers to the visits. By using a qualitative approach, we were able to unearth deeper factors that underlie the decisions to make the health visits, which in turn would influence allocation of the meagre financial resources by the families in facilitating the visits.

### ANC visits

The decision to make ANC visit was carefully considered, with the perceived benefits weighed against the constraints of time, opportunity cost, fatigue coupled with long walking distance required, lack of good clothes to wear amongst others. A decision would then be made on whether to make the visit. While previous studies have captured some of the latter components as challenges of making health visits [17, 18, 34], they have rarely been considered against the perceived benefits of attending the visits to the woman, and whether in their mind the benefits outweigh the cost. In this study we found that the perceived benefits of ANC attendance were biased towards treatment interventions, and less on preventive measures that do not entail a medical component, for instance education and counselling. Therefore, women who had difficult pregnancies and illnesses during pregnancy were more likely to attend ANC than those who did not experience any visible problems. For this latter group of women, the constraints greatly outweighed the perceived benefit, and therefore the decision to make the visit was deferred until late into the pregnancy when there was a more compelling reason to make the visit. This included the need to obtain ANC card as an insurance for facility delivery as has also been shown in other studies [25, 26]; or to have a scan to check on the lying position of the baby to prepare for complications during the delivery. We also found that deferral of ANC visits to late pregnancy resulted into a less likelihood of the visits being made at all. Deterrents to the visit such as tiredness, lack of appropriate transport mode and lack of appropriate clothes to wear become even more prominent in advanced stages of pregnancy.

We also found that lack of ANC attendance impacted on subsequent facility visits for delivery and postnatal care. Women who did not attend ANC feared reprimand from health workers hence would choose to deliver at home; while women who delivered at home skipped postnatal facility visits for fear of being reprimanded for home delivery. Several studies have linked ANC visits to facility delivery [18], but have not gone in-depth to explore why this is the case. The vast majority of the studies have been cross-sectional in nature and have overwhelmingly relied on data from household surveys [18, 27]. Our study confirms the findings of these previous studies but goes beyond them to explain factors underlying the low attendance witnessed, and the impact of this on subsequent health visits.

### Facility delivery

The unpredictability of labour emerged as the biggest deterrent to facility delivery. As expected, most labour was reported to begin at night, at a time when there would be no means of transport to the health facilities, therefore “forcing” the women to deliver at home. Most research to date that highlight distance and transport as deterrents to facility visits [27–29] have not brought forward clearly the connection with the timing of labour; and consequently there have been narrowly framed interventions that focus only on the transportation costs. What our findings suggest is a need for an advance plan for transportation to the health facility. Currently, the ANC interventions include preparation of a birth plan that also includes transportation planning. However, this does not quiet address the challenge even for women who attend ANC. The Expected Delivery Date (EDD) estimate is based on women’s recall of their last monthly period, which is often highly inaccurate, leading to inaccurate EDD estimates. Women who had gone for ANC in their previous deliveries reported that their delivery occurred very far from the estimated dates they were given at the facilities. Better ways of estimating EDD could thus be an avenue for intervening on facility delivery. Facility closures during the night and weekends considerably increased the distance that had to be travelled beyond what had been planned and restricted the transportation modes available. There is an urgent need for re-consideration of the opening times of the health facilities in this setting where transportation means is not available during night times.

Another important deterrent to facility delivery was lack of ANC attendance. This finding is consistent with those of other studies that show ANC attendance as a strong predictor of facility delivery [30]. Our study can explain this connection, in terms of fear of reprimand from facility staff for not going to ANC. This suggests a need for changing attitudes of the health workers. The women should be made to feel free to enter the healthcare system at any stage of their pregnancy and not be reprimanded for not having made visits earlier or turned away. The negative attitude of health workers towards women that go to deliver at health facilities also needs changing; as the experiences are shared with the woman’s social network who in turn choose to deliver at home or influence others to do so. Although there is no one-fits-all solution, performance-based incentives such as payment per number of deliveries has worked well in other settings [38] and was anecdotally suggested by some nursing staff we interacted with during the study.

Prior experience of a successful home delivery, health worker’s gender and attitude, fear of medical procedures and financial constraints were also deterrents to facility delivery, consistent with findings of other studies in similar settings [27, 31, 32]. These were weighed against the caring attitude of traditional birth attendants, familiarity of the home environment and availability of family support when one delivers at home. A systematic review of qualitative studies on facilitators and barriers to facility-based delivery [19] found that the emphasis placed on facility-based deliveries by public health entities has led women and their families to believe that childbirth has become medicalized and dehumanized. Participants in our study demonstrated a lack of understanding of most of the medical procedures. They referred to the health facilities as “those places”, and many interpreted lifesaving interventions like assisted birth as unnecessary and punitive, including participants with high risk pregnancies. Furthermore, the negative experience of one woman would be told and re-told and would influence not only her future decision to deliver at home, but also the decision of other women who heard the tale, and others in the family who had an influence on a woman’s choice of a delivery place. The influence of a woman’s social network on her choice of delivery place has been confirmed in other qualitative studies [33]. There is need for better communication between the health personnel and the clients, so that the benefits of facility delivery and the associated procedures are understood, and they do not in themselves become barriers.

### Postnatal visits

Compared to other health visits whose importance the women could articulate, participants could not articulate the importance of postnatal visits. For some women, the visit was unnecessary because the delivery milestone had already been achieved.

Multiple and complex barriers to postnatal visit were narrated. These included the health status of the woman which made it impossible to make the visit. Any intervention aimed at improving health visit for postnatal care should not overlook the major challenge for the women of having to walk to the distantly located health facilities while still sick and recovering from child birth. Even with availability of finances, the mode of travel available was stated as inappropriate for making the journey with a baby while still in a state of recovery.

Non-transport related challenges were also stated; such as fear of embarrassment, fear of reprimand from health staff for missed visits and fear of vaccinations. These challenges could be addressed through changing the attitude of health workers to make the women feel welcome at any stage of the maternal and child health care continuum; and to have better communication that is not perceived as reprimand. Better communication is needed not just with the women but also with the spouses and the entire community, so that the benefits of postnatal care and vaccinations are understood and internalised. Unlike the ANC and facility delivery, the spouses and relatives (mothers-in-law) had a strong influence on the woman’s decision to go for postnatal care.

The finding that home delivery is a deterrent to postnatal visits offers a strong suggestion that intervening on ANC attendance can lead to positive outcomes along the entire continuum of care to include facility delivery and postnatal visits.

### Strengths and limitations

The strengths of this study included an application of a qualitative approach, thereby addressing the limitations of many previous studies that have used quantitative survey methods. Household surveys are not ideal for measuring social norms and attitudes that influence decision-making patterns on healthcare seeking during pregnancy and childbirth. The study also assessed the barriers along the entire continuum of maternal care and was therefore able to identify key linkages and relationships between the three stages and the important areas of intervention. An additional strength of the study is that it interviewed women who were still in the process of making the health visits; hence were able to share their most recent experiences with the health visits, and decisions they had recently made or were about to make. In many studies, data are collected from women several months after delivery, requiring participants to reflect on the reasons that compelled them to make the visits. While this may be the most practical and feasible way to gather such information, these type of studies are subject to recall bias [33].

The limitation is that the women recruited were aware of a planned intervention and could have perhaps overstated the challenges, knowing that our aim was to design a programme that is responsive to these challenges. However, our findings are consistent with those of other studies in similar settings, suggesting that the effect of the planned intervention on our findings was negligible.

A general limitation of qualitative studies like ours is that they are context specific, and results can therefore not be generalised to other contexts. We therefore recommend similar studies in other settings that can help to inform public health policy in sub-Saharan Africa where maternal mortality remains unacceptably high, and urgent interventions are required to move towards the SDGs target. Future studies should also investigate ways of overcoming the challenges we have highlighted in this study.

## Conclusions

This study has shown that barriers to healthcare visits for maternal care are multiple and complex, therefore single focused interventions such as user fee removal that has been implemented in Kenya cannot fully tackle them. Broader based approaches are needed that can help overcome the multiple challenges. The study has also identified a strong need for education and awareness on the benefits of ANC attendance that go beyond routine treatment of medical conditions. These benefits would act as a catalyst for ANC attendance despite the financial challenges. Focusing on early ANC attendance is likely to have trickle up effects that include facility delivery and postnatal care. Education should not be restricted to women but to the whole family and community as they have a strong influence on a woman’s healthcare seeking decision. The health workers should be educated and incentivised on shifting their attitudes on how they interact with their clients, to allay the pervasive fear of reprimand that has a negative effect of deterring health visits for facility delivery and postnatal care.

## Additional file

Additional file 1: Question guide. (DOCX 19 kb)

## Data Availability

The datasets generated during and/or analysed during the current study are available from the corresponding author on reasonable request.

## Abbreviations

ANC: Antenatal Care
CCT: Conditional Cash Transfers
EDD: Estimated Delivery Date
FGD: Focus Group Discussions
HIV: Human Immunodeficiency Virus
MDG: Millenium Development Goal
SDG: Sustainable Development Goal
SWAP: Safe Water and AIDS Project
UNICEF: United Nations Children’s Fund
WHO: World Health Organisation
