# Supplementary material for: Barriers to formal health care seeking during pregnancy, childbirth and postnatal period: a qualitative study in Siaya County in rural Kenya
Source: BMC Pregnancy Childbirth. 2019 Sep 18;19:339. doi: 10.1186/s12884-019-2485-2 (PMC6751879; doi:10.1186/s12884-019-2485-2)
Supplement: Supplementary file 1 — Question guide. (DOCX 19 kb) [file 12884_2019_2485_MOESM1_ESM.docx]

**Barriers to formal health care seeking during pregnancy, childbirth and postnatal period: A qualitative study in Siaya County in Kenya**

FGD Guide

1. **What are or would be the main barriers for you in attending antenatal care clinic?**
2. **What are or would be the main barriers for you in giving birth at a health facility?**
3. **What are or would be the main barriers for you in bringing back your newborn for postnatal visit (within 30 days of delivery?)**

***Probes:***

*What do you think about before deciding whether or not to go for ANC visit? Hospital delivery? Postnatal visit?*

*Is the decision made solely by your, or your family and household have a say in the matter?*

*What makes you miss the visits – ANC? Hospital delivery? Post-natal follow-up?*

*Are these barriers in any way income related, directly or indirectly?*

*How often was it because you thought you did not need care?*

*How often was it because you were not feeling well?*

*Lack of transportation?*

*Busy?*

*Forgot?*

*Fear of others learning about your pregnancy and fear of stigma?*

*Anything else?*
